# Exploring the impact of protected fat on fattening performance, carcass characteristics, fatty acid composition, and meat quality in Tuj lambs

**DOI:** 10.5194/aab-68-183-2025

**Published:** 2025-03-07

**Authors:** Dogan Turkyilmaz, Ulku Dagdelen, Nurinisa Esenbuga, Seyma Sisik Ogras

**Affiliations:** 1 Department of Animal Science, Faculty of Agriculture, Atatürk University, Erzurum 25240, Türkiye; 2 Department of Food Engineering, Faculty of Agriculture, Atatürk University, Erzurum 25240, Türkiye

## Abstract

The study assessed the impact of incorporating protected tallow fat (PF) at varying concentrations on the fattening performance, carcass characteristics, meat quality, and fattening cost of 45 lambs over a period of 73 d. The addition of 20 g per kg dry matter (DM) of PF resulted in the highest values for fattening performance and carcass characteristics ( p<0.05 ). The pH, chemical composition, and sensory evaluation were not affected by the addition of PF, except for the cooking loss ( p<0.05 ). The addition of PF led to an improvement in the sums of fatty acids in the longissimus thoracic muscle by reducing saturated fatty acids (SFAs) and increasing unsaturated fatty acids (UFAs). The addition of PF at a level of 20 g per kg DM positively affected ( p<0.05 ) the ratio of oleic acid, one of the major UFAs in meat. The ratio of conjugated linoleic acid (CLA) in meat increased linearly up to 104 %, with increasing ( p<0.05 ) levels of PF. The healthy indicators considerably improved ( p<0.01 ). In conclusion, it is recommended to add PF at a 20 g per kg DM inclusion level to the mixed concentration to improve fattening performance, carcass characteristics, meat quality, and fatty acid composition without any economic loss.

## 1 Introduction

Nutrition strategies have been developed in sheep farms to increase the efficiency of production, particularly milk and meat, and various feeds are used for this purpose (Liu et al., 2023). The strategies of the native sheep have gained more importance due to an important factor limiting the production, that is, the low-producing potentials. For native sheep in Türkiye, the current production performances are not sufficient. Sheep meat production can be increased by improving the growth rate and feed conversion efficiency of genetically improved Turkish breeds with nutrition strategies (Macit et al., 2001). Besides, the meat quality can likewise be developed with the strategies. Despite the biohydrogenation of unsaturated fatty acids (UFAs) in the rumen, lipid supplementation to ruminant diets is the most effective method of providing positive benefits to the fatty acid composition of their products (Chikwanha et al., 2018).

Lamb meat is an important source of conjugated linoleic acid (CLA), n-3 fatty acids, and other unsaturated fatty acids (UFAs), and it is rich in saturated fatty acids (SFAs). The ratio of SFA to UFA is measured using a number of indices and evaluated in terms of health such as the risk of vascular and coronary diseases (Barahona et al., 2016). Likewise, foods containing fatty acids low in the ratio of poly-unsaturated fatty acids (PUFAs) to SFAs and high in the ratio of n-6 to n-3 cause unbalanced fatty acid intake in human diets. Healthier products, which are associated with general well-being, are preferred by consumers (De Smet and Vossen 2016). It has caught consumers' attention that CLA, particularly c9, t11 and t10, c12 isomers, have healthy nutritional effects. Thus, there has been a tendency to improve the fatty acid content of meat by modifying the diet (Scollan et al., 2017). The fat content of meat affects the physical and chemical traits of the meat products (Wood et al., 2008). Consumers pay attention to sensory and visual characteristics when preferring quality meat as well as the fat content (Sousa et al., 2022).

There are PUFAs, including CLAs, obtained from the biohydrogenation of UFAs by the microbiota in the rumen (Sousa et al., 2022). However, a substantial proportion of the UFA from dietary lipids is saturated by hydrolysis and biohydrogenation and passes into the duodenum and tissues as SFA. Dietary lipids can be protected with calcium soaps to improve meat quality by increasing the proportion of health-important fatty acids in meat by bypassing UFAs from biohydrogenation in the rumen (Salinas et al., 2006; Bhatt et al., 2020).

Numerous studies have been carried out to improve lamb productive performances, such as carcass traits and meat quality. However, these studies have mostly used protected or unprotected vegetable oil supplementation compared to tallow-fat sources. The production of calcium soaps from tallow is cost-effective and similar to the process used for palm-oil-derived calcium soaps. Tallow-based calcium soaps have a granulated–solid structure that allows easy blending with ration components. This provides increased resistance to oxidation and offers the recognized digestive and physiological benefits associated with rumen-protected lipids in animal nutrition (Salinas et al., 2006). The production of calcium soap from tallow involves a saponification reaction, where tallow reacts with calcium hydroxide, resulting in the formation of granulated–solid calcium soaps and glycerol. After separation and purification processes, the dried calcium soaps become suitable for incorporation into animal feed. The tallow's calcium soaps protect fatty acids from biohydrogenation, allowing some unsaturated fatty acids to bypass microbial activity in the rumen and pass into the small intestine. This enhances the absorption and utilization of unsaturated fatty acids by the ruminant for energy and physiological functions.

The study hypothesizes that supplementing the diet of lambs with tallow fat characterized by high unsaturated fatty acids and protected with calcium soap will lead to a discernible improvement in meat quality, including nutritional composition, such as fatty acid content, and sensory attributes. The economic impact of the results obtained was also assessed. The objective of the study was to determine the effects of protected tallow fat supplemented to the ration on fattening performance, carcass traits, meat quality, and fatty acid composition of Tuj lambs.

## 2 Material and methods

### 2.1 Animals, diets, and facilities

Forty-five 8-month-old fat-tailed Tuj lambs were kept in pens within a feedlot that had a covered area and shade for food, at the Sheep Farm of Food and Livestock Animals Application and Research Centre (GHUAM) at Atatürk University, Erzurum, Türkiye. The lambs were fattened for 73 d of the experiment after 14 d of adaptation to the experimental diet and feedlot conditions. After the adaptation period, the lambs were introduced to the experiment. The lambs, which had similar live weights, were divided into three groups and were fed together. The control group was fed ad libitum concentrate feed, while the remaining two groups received ad libitum concentrate feed with 20 g per kg dry matter (DM) protected fat and 40 g per kg DM protected fat (Table 1). The study used tallow obtained from a private company's production as the protected fat. The tallow is characterized by high content of unsaturated fatty acids and protected by calcium soaps (Table 2). The protected fat is homogeneously mixed into the concentrated feed. The tallow protected with calcium soaps had 85 % crude fat, 8 % calcium, 4 % moisture, and energy of 7228 kcal per kg metabolizable energy (ME). The daily feed conversion rate of the lambs was recorded. Lambs' live weight was recorded every 14 d until the day before they were slaughtered.

**Table 1:** Formulation and chemical compositions of the diets of groups.

Item	Protected tallow-fat inclusion		
	[g per kg DM]		
	0	20	40
Ingredient [g per kg DM of diet]			
Barley	464.3	454.9	445.5
Meadow grass	285.8	279.8	274.1
Soybean meal	157.1	154.0	150.8
Wheat bran	78.6	77.0	75.4
Dicalcium phosphate	7.1	7.0	6.9
Salt	7.1	7.0	6.9
Ca soaps of fatty acid	–	20.3	40.4
Chemical composition [%]			
Dry matter	87.9	88.0	88.1
Crude fiber	17.8	17.3	17.4
Crude protein	12.4	12.4	12.1
Starch	27.0	26.7	26.0
Ash	9.0	8.9	8.8
Ether extract	2.4	3.3	4.4
Ca	0.26	0.34	0.45
ME [ $\mathrm{kcal}\;{\mathrm{kg}}^{-1}$ ]	2530	2600	2640

ME: metabolizable energy, DM: dry matter.

**Table 2:** Fatty acid composition of experimental diets.

Item	Concentrated mix	Protected tallow fat
Fatty acid composition [g per 100 g fatty acids]		
C14:0	0.17	3.09
C14:1	–	0.33
C16:0	10.04	27.51
C16:1	–	1.87
C17:0	–	1.22
C18:0	2.47	22.36
C18:1n9c	27.16	36.36
C18:2n6c	53.95	3.90
C18:3n3	3.93	–
C20:1	0.28	–
C20:3n3	0.38	–
C20:5n3	0.58	–
C22:0	0.12	0.18
C22:6n3	0.09	–
Others	0.83	3.18
SFA	13.30	57.06
MUFA	27.94	39.02
PUFA	58.75	3.90

SFA: saturated fatty acid, MUFA: mono-unsaturated fatty acid, PUFA: poly-unsaturated fatty acid.

### 2.2 Slaughtering and tissue sampling

Eight lambs from each group were randomly selected and slaughtered at a commercial slaughterhouse following a 12 h fast for three consecutive days. The eight animals selected for the experiment were determined by the Power-G analysis method, which considered factors such as animal welfare and cost-effectiveness. The average weight recorded on these three days was determined as the slaughter weight. The lambs were slaughtered, and the inedible parts, such as offal, feet, tail, skin, and head, were separated. The separated parts were weighed. The hot carcass weight (HCW) was recorded before halving the carcass. The half-carcasses were then stored in a chilled chamber at 4 °C for 24 h, after which the weight of the cold carcass was determined by weighing the left side of each carcass. The commercial categories for the left-half sides were divided based on anatomical regions, such as the rib and loin, forearm, leg, breast, and neck. Quantitative assessments were conducted to measure the surface area of the longissimus thoracic (LT) muscle and the fat thickness. Measurements were taken from the transverse section between the 12th and 13th ribs, extending laterally to the vertebral column. The carcasses were evaluated for marbling, yield grade, and proportion of boneless retail cuts (Boggs and Merkel, 1984). Marbling was quantitatively evaluated by assessing the intramuscular fat deposition in the LT muscle, utilizing predefined standards and employing a numerical scoring system, where scores ranged from 1 (trace) to 6 (abundant).

### 2.3 Meat quality measurements

The samples were stored at a temperature of +4°C and subsequently transferred to the Laboratory of the Department of Animal Science at Atatürk University. Before meat quality measurements, samples were stored at –20 °C . The frozen samples were then gradually thawed for analysis.

The pH levels of the LT muscles were determined by direct probe measurements using a SCHOTT Lab Star pH meter on freshly cut surfaces. Color parameters of the LT muscles were assessed 24 h postmortem at identical locations and following 30 min of exposure to air. A Minolta colorimeter device (CR-400) was employed for the objective quantification of CIELAB brightness ( $L^{*}$ ), green–red color ( $a^{*}$ ), blue–yellow color ( $b^{*}$ ), hue angle ( H ), and chrome ( C ) values. The evaluation of dry matter, ether extract, and crude protein content in the LT muscle was conducted in adherence to AOAC (1999).

The meat samples were cooked in a water bath at 90 °C until the internal temperature reached 70 °C for sensory evaluation. The samples were divided into two subsamples, one for weight assessment to determine cooking losses and measure Warner–Bratzler shear force (WBS) and the other for sensory panel evaluation. The cooking loss and WBS analysis were conducted in accordance with the protocol by Turkyilmaz and Esenbuga (2022), as modified by the guidelines established by AMSA (2015). The weight losses resulting from the cooking process were determined by measuring the samples before and after cooking, and the differences between these weights were calculated. The results obtained were then used to express the cooking losses. Subsequently, the samples were cut with a 12.7 mm cylinder, a hollow punch, and an industrial drill. Then, the Warner–Bratzler shear force blade, equipped with a 60° angled cutting edge, was used to determine the WBS. The cutting process was recorded, and the WBS was determined. The cutting process was recorded, and the WBS was determined in kilograms ( $\mathrm{kg}\;{\mathrm{cm}}^{-3}$ ) as the average of six values per sample, with the lowest and highest values of the measurement removed to ensure the reliability of the results. Sensory analysis was conducted through panelists' ratings of flavor (from intense mutton flavor to intense lamb flavor), tenderness (from extremely tough to extremely tender), juiciness (from extremely dry to extremely juicy), and overall acceptability (from extremely low acceptability to extremely high acceptability) on a nine-point hedonic scale. The panel members also determined the number of chews before swallowing.

Total lipid extraction and transmethylation procedures described by Türkyılmaz and Esenbuga (2022) were performed to determine the fatty acid composition of intramuscular adipose tissue of longissimus thoracic (LT) muscles. The quantification of fatty acid methyl esters (FAMEs) within the intramuscular adipose tissue was carried out using gas chromatography (GC) on a Perkin Elmer Clarus 500 instrument equipped with a flame ionization detector (FID). A capillary column, specifically CP-Sil 88, measuring 100 m in length, with a 0.25 µm inner diameter, and featuring a 0.20 µm thick film was utilized. Helium, maintained at a flow rate of 1.0 $\mathrm{mL}\;\min^{-1}$ , served as the carrier gas. The oven temperature program was set to between 70 and 240 °C .

The fatty acid composition determination in this study involved a meticulous process. Each sample (1 µL ) was injected into the chromatograph using an auto-sampler. To align retention times for individual fatty acids and ensure accuracy in our assessments, we used the Supelco™ Component FAME Mix chromatography standard (category no. 18919, Supelco, Bellefonte, PA). The fatty acid content was quantified by expressing peak areas in the chromatogram as a percentage relative to the total fatty acid content (g per 100 g of total fatty acids). Key indices, including the atherogenicity index (AI) and thrombogenicity index (TI), were also calculated using the well-established methodology outlined by Ulbricht and Southgate (1991). The activity of $\Delta^{9}$ -desaturase (C16 and C18) was assessed using the protocol proposed by De Smet et al. (2004). Additionally, the elongase index and hypocholesterolemic / hypercholesterolemic (h / H) ratio were calculated following the procedures outlined by Santos-Silva et al. (2002).

### 2.4 Economic assessments

The gross profit analysis, taking into account revenues and expenses, was determined based on live and carcass sale weights. The economic assessments were made using lamb prices obtained by GHUAM; feed costs (including concentrate and protected fat) by a commercial company; and carcass prices by the National Red Meat Council (UKON), a Turkish organization, at the time of the study.

### 2.5 Statistics

The experimental design was completely randomized using 45 lambs and three levels of protected fat. The general linear model of the SPSS (2013) package was used for statistical analysis. A mathematical model that accounted for the effects of protected fat at different levels was used to analyze data on fattening performance, slaughter traits, meat quality, and fatty acid composition. The number of slaughtered lambs was determined by performing a Power-G analysis, considering animal welfare. The mathematical model is

1 $$Y_{ij}=\mu +\alpha_{i}+\beta (X_{ij}-\bar{X})+e_{ij},$$

where $Y_{ij}$ is the dependent variable value observed, μ is the overall average of the treatments used in the study, $\alpha_{i}$ is the fixed effect of diet ( i : 0, 20 and 40 g per kg DM), $\beta (X_{ij}-\bar{X})$ is the effect of covariance, and $e_{ij}$ is the error of the experiment. Polynomial analysis was used to determine whether the increase in protected fat levels in the diets tended towards a linear ( L ) or quadratic ( Q ) trend. The Kruskal–Wallis test was used to verify the statistical analysis of sensory properties. Duncan's multiple range test was used to assess statistical differences between means.

## 3 Results

Mean values of fattening performance variables for Tuj male lambs are shown in Table 3. Average daily weight gain ( p<0.01 ) and final weights ( p<0.05 ) were significantly higher between groups with similar initial weights. In line with this increase, the feed conversion ratio was significantly affected ( p<0.01 ) by the protected fat addition, although the dry matter intake of all lamb groups was similar.

**Table 3:** Effects of protected fat on fattening performance on Tuj lambs.

Variable	Protected tallow-fat inclusion			SEM	P
	[g per kg DM]				
	0	20	40		
Fattening performance [kg]					
Initial weight	29.455	30.799	30.102	1.295	0.763
Final weight	42.273^b^	48.636^a^	45.889^a,b^	1.403	0.020
Average daily gain	0.193^b^	0.261^a^	0.248^a^	0.008	< 0.001
Dry matter intake	1.18	1.35	1.25	0.139	0.787
Feed conversion*	6.13^b^	5.16^a^	5.05^a^	0.176	< 0.001

^a,b^ Means in rows with different superscripts are significantly different. ^*^ The amount of feed consumed for 1 kg of live weight gain. DM: dry matter, SEM: standard error of means, P : significance value.

The addition level of protected fat affected most of the carcass characteristics, except for the carcass quality indicators (Table 4). The carcass weights were increased by about 15 % in 20 g per kg added group ( p<0.05 ), while the lambs with an added 40 $g\;{\mathrm{kg}}^{-1}$ were found to be similar to the control group. Although the dressing of lambs fed with the added fat was higher than the control group, the differences between groups were statistically insignificant. The lambs with the highest percentage of tail fat were the control group ( p<0.01 ). However, there were no significant differences between groups in terms of the tail fat weight.

**Table 4:** Effects of protected fat on slaughter traits and carcass characteristics of Tuj lambs.

Variable	Protected tallow-fat inclusion			SEM	P
	[g per kg DM]				
	0	20	40		
Slaughter traits [kg]					
Slaughter weights	46.82^b^	51.86^a^	46.72^b^	1.698	0.031
Hot carcass	22.08^b^	25.58^a^	22.48^b^	0.902	0.035
Cold carcass	21.80^b^	25.14^a^	22.12^b^	0.913	0.045
Dressing [%]	46.46	48.27	47.33	0.345	0.691
Feet	0.949	0.953	0.886	0.029	0.229
Head	2.648	2.809	2.652	0.103	0.472
Skin	5.822	6.183	5.518	0.446	0.587
Offal	1.609	1.708	1.576	0.057	0.270
Testicle	0.327	0.333	0.259	0.035	0.288
Kidney	0.159	0.184	0.181	0.019	0.603
Carcass components [kg]					
Forearm	3.24	3.52	3.06	0.123	0.057
Leg	6.50^b^	7.54^a^	6.44^b^	0.284	0.029
Neck	3.06	3.48	3.16	0.152	0.171
Rib and loin	2.70^b^	3.00^a,b^	3.32^a^	0.109	0.006
Breast	2.87^b^	3.61^a^	2.95^b^	0.150	0.009
Tail fat	3.33	3.89	3.10	0.167	0.119
Carcass components [%]					
Forearm	14.91^a^	14.13^b^	13.81^b^	0.120	< 0.001
Leg	29.78^b^	30.25^a^	29.09^c^	0.137	< 0.001
Neck	14.01	13.92	14.26	0.140	0.242
Rib and loin	12.39^b^	12.06^b^	15.01^a^	0.170	< 0.001
Breast	13.17^b^	14.45^a^	13.32^b^	0.148	< 0.001
Tail fat	16.10^a^	15.56^b^	15.24^b^	0.159	0.007
Carcass quality					
Marbling score	4.0	3.5	3.4	0.44	0.596
LT area [ ${\mathrm{cm}}^{2}$ ]	16.5	16.7	16.9	1.41	0.659
Fat thickness [mm]	8.0	7.2	7.8	1.60	0.935
Yield grade	3.6	3.2	3.5	0.62	0.930
Retail cut [%]	44.4	45.0	44.6	1.10	0.934

^a,b^ Means in rows with different superscripts are significantly different. SEM: standard error of means, P : significance value, SEM: standard error of means, LT: longissimus thoracic.

The pH values in all lamb groups were found to be between 5.4 and 5.6, as it should be in meat for consumption (Sanudo et al., 2007), and there was no significant change from the 24th hour to the 120th hour (Table 5). Likewise, chemical compositions such as crude protein, ether extract, and dry matter were not influenced by the inclusion of protected fat in the diet.

**Table 5:** Effects of protected fat on pH, color, and chemical composition of LT muscles.

Variable	Protected tallow-fat inclusion			SEM	P	
	[g per kg DM]					
	0	20	40			
24th hour						
pH value	5.47	5.54	5.61	0.054	0.215	
$L^{*}$ (lightness)	34.01	33.93	34.72	0.394	0.306	
$a^{*}$ (redness)	14.54	14.84	15.15	0.348	0.470	
$b^{*}$ (yellowness)	4.21	4.48	4.41	0.372	0.867	
Hue angle	15.20	15.57	15.84	0.357	0.974	
Chrome value	16.19	16.59	16.23	1.343	0.456	
72nd hour						
pH value	5.49	5.55	5.54	0.032	0.371	
$L^{*}$ (lightness)	35.38	33.62	34.45	0.492	0.052	
$a^{*}$ (redness)	16.50	15.04	15.69	0.418	0.056	
$b^{*}$ (yellowness)	4.78^a^	3.98^b^	4.29^a,b^	0.217	0.039	
Hue angle	17.21	15.58	16.28	0.431	0.277	
Chrome value	16.26^a^	14.72^b^	15.22^a,b^	0.681	0.036	
120th hour						
pH value	5.48	5.55	5.53	0.026	0.135	
$L^{*}$ (lightness)	35.14	33.86	34.43	0.535	0.251	
$a^{*}$ (redness)	15.84	16.26	17.95	1.366	0.521	
$b^{*}$ (yellowness)	3.98	4.06	4.05	0.218	0.967	
Hue angle	16.34	16.76	18.44	1.365	0.642	
Chrome value	14.09	14.00	13.36	0.599	0.525	
Chemical composition [g per 100 g LT muscle]						
Crude protein [%]	21.93	21.60	21.87	0.574	0.909	
Ether extract [%]	3.15	3.10	3.68	0.260	0.259	
Dry matter [%]	25.38	24.76	24.92	0.515	0.688	

^a,b^ Means in rows with different superscripts are significantly different. SEM: standard error of means, P : significance value, LT: longissimus thoracic.

Sensory evaluations were an important trait in terms of consumer satisfaction (Warriss, 2000). The results in the study showed that the addition of protected fat in the diet did not affect the evaluation scores for different sensory traits (Table 6).

**Table 6:** Effects of protected fat on sensory evaluation of LT muscles.

Variable	Protected tallow-fat inclusion			SEM	P
	[g per kg DM]				
	0	20	40		
Tenderness	5.7	5.4	5.9	0.24	0.345
Juiciness	5.4	5.6	6.1	0.24	0.137
Flavor	5.8	5.9	5.4	0.22	0.262
Overall acceptability	5.8	5.6	5.7	0.22	0.758
The number of chews	25.2	23.2	22.7	1.85	0.121
Cooking loss [%]	25.6	23.7	23.8	1.45	0.090
WBS [ $\mathrm{kg}\;{\mathrm{cm}}^{-2}$ ]	4.02	4.05	4.12	0.336	0.976

WBS: Warner–Bratzler shear force, P : significance value, SEM: standard error of means, LT: longissimus thoracic.

The composition of fatty acids contributes to important aspects of meat quality and plays an important role in improving its functional value (Webb et al., 2022). The fatty acid composition (% of total fatty acid) of lambs fed different levels of protected fat is shown in Table 7. As increasing protected fat level in the diets, C18:1n9c (oleic acid), C18:2n6c (linoleic acid), and C18:3n6 (GLA) increased in a quadratic fashion ( p<0.05 ), while C16:0 (palmitic acid) and C18:0 (stearic acid) tended to decrease although not statistically significantly. The groups with added protected fat in the diet had a lower ( p<0.05 ) proportion of C14:0 (myristic acid) compared to the control diet. Also, the addition of protected fat to the lamb diet led to a linear increase ( p<0.05 ) in C18:1n9t (elaidic acid) and CLA (c9, t11). The content of C18:3n3 ( α -linolenic acid, ALA), C20:5n3 (eicosapentaenoic acid, EPA), and C22:6n3 (docosahexaenoic acid, DHA), which are the main n-3 fatty acids, were found to be similar between groups.

**Table 7:** Effect of protected fat on fatty acid composition of LT muscles.

Content	Protected tallow-fat inclusion			SEM	P	L	Q
[g per 100 g of total fatty acids]	[g per kg DM]						
	0	20	40				
C10:0	0.162	0.112	0.126	0.026	0.396	0.343	0.331
C12:0	0.308	0.236	0.476	0.101	0.262	0.261	0.230
C13:0	0.072	0.084	0.066	0.009	0.374	0.641	0.192
C14:0	16.072^a^	9.782^b^	10.980^b^	1.255	0.009	0.014	0.031
C14:1	1.358	1.734	1.346	0.193	0.306	0.966	0.132
C15:0	0.102	0.092	0.090	0.026	0.940	0.747	0.901
C15:1	1.220	1.110	1.330	0.225	0.791	0.736	0.561
C16:0	19.130	18.872	17.602	1.187	0.633	0.381	0.734
C16:1	0.422	0.398	0.292	0.076	0.456	0.247	0.665
C18:0	18.108	17.876	16.834	0.476	0.156	0.061	0.818
C18:1n9t	2.834^b^	2.070^b^	4.748^a^	0.575	0.018	0.037	0.031
C18:1n9c	24.130^b^	30.642^a^	25.784^b^	1.619	0.037	0.484	0.014
C18:2n6t	1.482	1.318	1.384	0.105	0.557	0.523	0.390
C18:2n6c	3.590^b^	5.620^a^	4.196^b^	0.352	0.005	0.247	0.002
c9, t11 CLA	0.582^b^	0.604^b^	1.186^a^	0.144	0.019	0.012	0.138
t10, c12 CLA	0.288	0.230	0.392	0.057	0.169	0.222	0.141
C18:3n3	0.428	0.544	0.658	0.117	0.411	0.191	0.995
C18:3n6	0.128^b^	0.236^a^	0.174^ab^	0.031	0.087	0.318	0.046
C20:2	0.074	0.052	0.048	0.010	0.187	0.093	0.479
C20:3n3	0.208	0.308	0.326	0.054	0.288	0.148	0.547
C20:3n6	0.058	0.068	0.036	0.021	0.565	0.476	0.434
C20:4n6	2.856	2.280	2.336	0.348	0.459	0.312	0.473
C20:5n3	0.204	0.320	0.258	0.059	0.408	0.530	0.242
C22:2	0.444^a^	0.114^b^	0.174^b^	0.078	0.024	0.030	0.063
C22:5	0.330	0.546	0.480	0.142	0.560	0.469	0.433
C22:6n3	0.168	0.180	0.160	0.056	0.968	0.921	0.818
C23:0	0.238	0.180	0.176	0.039	0.482	0.288	0.586
C24:0	0.030	0.026	0.028	0.007	0.918	0.839	0.726
C24:1	0.074	0.058	0.070	0.011	0.576	0.801	0.317
Others	6.054	5.652	5.746	0.143	0.281	0.209	0.323

^a,b^ Means in rows with different superscripts are significantly different. SEM: standard error of means, P : significance value, L : linear, Q : quadratic, DM: dry matter, CLA: conjugated linoleic acid, LT: longissimus thoracic.

The study showed that adding protected fat at both levels increased the MUFA content and decreased the SFA content (Table 8). Lambs fed both levels of protected fat had decreased SFAs and increased MUFAs ( p<0.05 ), while PUFA levels remained unchanged. Although the 20 g per kg added group had a significantly higher MUFA / SFA ratio ( p<0.05 ), the desired increase in the PUFA / SFA ratio was not observed. The thrombogenicity index (TI) and atherogenicity index (AI), which are used to express the effects of fatty acid composition on human health (Ulbricht and Southgate, 1991), showed a significant positive effect of both levels of protected fat. was The $\Delta^{9}$ -desaturase C18 index showed a significant linear increase ( p<0.01 ). However, no changes were observed in the $\Delta^{9}$ -desaturase C16 index and elongase activity index. Also, the h / H index increased significantly ( p<0.05 ).

**Table 8:** Effect of protected fat on sums of fatty acids and healthy indicators of LT muscles.

Variable [%]	Protected tallow-fat inclusion			SEM	P	L	Q
	[g per kg DM]						
	0	20	40				
SFA	52.182^a^	46.476^b^	48.922^b^	0.958	0.004	0.033	0.005
MUFA	30.038^b^	36.012^a^	33.57^ab^	1.390	0.032	0.098	0.029
PUFA	11.446	11.86	11.762	0.901	0.944	0.808	0.821
MUFA / SFA	0.579^b^	0.776^a^	0.688^a^	0.034	0.006	0.046	0.006
PUFA / SFA	0.219	0.256	0.241	0.020	0.454	0.455	0.317
UFA / SFA	0.799^b^	1.032^a^	0.929^a^	0.037	0.003	0.029	0.003
n-3	1.65	1.53	1.99	0.332	0.625	0.486	0.510
n-6	10.93^a^	8.87^b^	7.90^b^	0.582	0.011	0.004	0.530
n-6/n-3	6.82^a^	5.97^a,b^	4.66^b^	0.584	0.063	0.022	0.746
AI	1.43^a^	1.01^b^	1.12^b^	0.071	< 0.001	0.005	0.006
TI	1.96^a^	1.67^b^	1.73^b^	0.047	< 0.001	0.002	0.006
$\Delta^{9}$ -desaturase C16	2.19	1.51	1.99	0.332	0.346	0.685	0.165
$\Delta^{9}$ -desaturase C18	58.43^b^	61.09^b^	66.02^a^	1.268	< 0.001	< 0.001	0.150
Elongase index	69.75	70.94	69.91	0.974	0.656	0.910	0.372
h / H	0.98^b^	1.29^a^	1.06^b^	0.068	0.017	0.406	0.006

^a,b^ Means in rows with different superscripts are significantly different. SEM: standard error of means, P : significance value, L : linear, Q : quadratic, DM: dry matter, LT: longissimus thoracic, SFA: saturated fatty acids, MUFA: mono-unsaturated fatty acids, PUFA: poly-unsaturated fatty acids, UFA: total unsaturated fatty acids, n-3: omega-3 fatty acids, n-6: omega-6 fatty acids, AI: atherogenic index, TI: thrombogenic index, $\Delta^{9}$ -desaturase C16 = [100 × (C16:1) / (C16:1 + C16:0)], $\Delta^{9}$ -desaturase C18 = [100 × (C18:1) / (C18:1 + C18:0)], elongase index = 100 × [(C18:1n9c + C18:0) / (C16:1 + C16:0 + C18:1n9c + C18:0)], h / H = (C18:1n9c + C18:2n6t + C18:3n3 + C20:4n6 + C20:5n3 + C22:5n3 + C22:6n3) / (C14:0 + C16:0).

The gross profit analysis for fattening with protected fat supplemented rations is shown in Table 9. Although total feed costs were higher in protected fat addition groups depending on the consumption and protected fat costs, the gross profit obtained from these groups was increased. On the other hand, lamb carcasses in the protected fat added groups had relatively higher sale revenues and gross profit. All groups had similar profit margins for carcass weights, but for live weight sales, the lambs with added protected fat in the diet had better results.

**Table 9:** Effects of supplementing protected fat on gross profit (USD) of Tuj lambs.

Variable	Protected tallow-fat inclusion			P
	[g per kg DM]			
	0	20	40	
Total lamb cost	95.2	97.8	95.5	0.895
Total feed cost	21.5^b^	25.0^a^	24.7^a^	< 0.001
Live weights sale				
Total revenue	136.1^b^	154.4^a^	150.3^a^	0.037
Gross profit	25.9^b^	40.9^a^	39.0^a^	< 0.001
Profit margin	0.18^b^	0.25^a^	0.25^a^	< 0.001
Carcasses sale				
Total revenue	142.3	162.7	144.3	0.068
Gross profit	35.5	49	44.6	0.156
Profit margin	0.04	0.05	0.05	0.338

^a,b^: Means in rows with different superscripts are significantly different. P : significance value, DM: dry matter.

## 4 Discussion

The lambs supplemented with protected fat showed the best feed conversion efficiency, with those in the 40 g per kg DM group consuming 18 % less feed per kg of weight gain. However, Gravador et al. (2020) reported that protected plant-derived products supplementation did not have an effect on fattening performance in Texel × Scottish Blackface crossbreed lambs.

The rib and loin weights, considered the most valuable part in terms of carcass components, were found to be higher in the lambs with an added 40 $g\;{\mathrm{kg}}^{-1}$ protected fat compared to other dietary groups. In many studies, feeding with added fat did not affect fat thickness, yield grade, and retail cut (Castro et al., 2005; Arana et al., 2006; Salinas et al., 2006; Kitessa et al., 2009; Mierlita et al., 2010; Bhatt et al., 2020), and similar results were found in this study.

The pH value is quite an important indicator for meat quality because it is affected by glycogenolysis in the muscle after the slaughter process and a subsequent increase in lactic acid, known as rigor mortis. The effect on the pH value leads to influence other parameters, such as color and sensory evaluations (Oliveira et al., 2014; Shange et al., 2018). After the slaughtering, pH values can usually drop to 5.2 due to rigor mortis in the muscles. This decrease in pH value points out the lactic acid accumulation. The results obtained in the study are similar to several other studies (Kitessa et al., 2009; Esenbuga et al., 2011; Castro et al., 2016).

The pH values show that the lambs were not exposed to stress before slaughter and that glycolysis did not increase after slaughter (Alba et al., 2021). The inclusion of protected fat in the diet did not adversely affect the pH value of the lamb meats. Therefore, no undesirable results were found in the color parameters.

In agreement with the findings of Gravador et al. (2020), sensory traits were not affected by the inclusion of protected fat in the diet. WBS value is a decisive factor in measuring tenderness. The meat with a WBS of less than 2.27 $\mathrm{kg}\;{\mathrm{cm}}^{-3}$ is evaluated as tender, between 2.27 and 3.63 $\mathrm{kg}\;{\mathrm{cm}}^{-3}$ is evaluated as medium, between 3.63 and 5.44 is evaluated as tough, and higher than 5.44 is evaluated as extremely tough (Valença et al., 2020). Consistent with studies on the inclusion of protected fat in the diet, WBS value was evaluated as tough in all lamb groups, and it was determined that the addition of protected fat had no effect on meat tenderness (Castro et al., 2016; Alba et al., 2021).

The intake of different levels of protected fat had a significant effect on the important fatty acids, especially on the total amount of fatty acids, in Tuj lamb meat. The content of CLA (c9, t11) linearly increased by 104 % with protected fat addition to the diet. This enhanced could be ascribed to the quadratic decrease in linoleic acid, which is conjugated by partial biohydrogenation in the rumen, increasing the addition level to 4 % in the diet. Contrary to long-standing criticism that animal fats promote chronic disease, there is increasing interest in CLA as a result of numerous animal studies attributing to CLA with beneficial health properties, such as improving immune function and reducing the risk of cancer, atherosclerosis, and diabetes (Webb and O'Neill, 2008; den Hartigh, 2019; Basak and Duttaroy, 2020).

However, as increasing protected fat levels in the diets, the content of major unsaturated fatty acids, including oleic acid and linoleic acid, exhibited a quadratic trend reaching the highest level in the 20 g per kg added group. A quadratic increase in MUFAs was observed as the addition level of protected fat increased, depending on insignificant changes in minor MUFAs and significantly changes in oleic acid and elaidic acid. The results show that oleic acid, linoleic acid, GLA, and partially myristic acid and EPA showed a quadratic increase rather than a linear increase, which, similarly to MUFAs, may be due to the optimal dose effect. The conversion of dietary fatty acids in the rumen and other gastrointestinal tissues could be affected by the intake dose. Statistically significant increases in elaidic acid and CLA, as well as marginal increases in myristic acid and EPA, may serve as indicators of this effect. The inclusion of protected fat in the diets exhibited neither a linear nor a quadratic effect on the contents of palmitic acid and stearic acid. Contrastingly to MUFAs, a quadratic decrease in SFAs was found despite insignificant differences in palmitic acid and stearic acid, the major SFAs, but this is probably due to the significant decrease in myristic acid, which is hypercholesterolemic and associated with cardiovascular disease and type 2 diabetes. In the studies that examined the effect of protected fat feeding on the fatty acid composition of lamb meat, it was found that there were no significant differences in the concentration of myristic acid, palmitic acid, and stearic acid (Elmore et al., 2005; Arana et al., 2006; Kitessa et al., 2009), while feeding with unprotected fat was reported to decrease them (Valençna et al., 2020; Alves da Costa et al., 2020), similarly to the present study.

Although there was no decrease in PUFAs, and even a marginal increase, with the addition of protected fat to the diet, no significantly increase was observed in the PUFA / SFA ratio as expected. The PUFA / SFA ratio was found to be below the recommended value of at least 0.4 by nutritionists. However, red meat generally has a ratio on the order of 0.1, and it can be deduced that the ratio obtained in the study is close to the desirable value (Webb and O'Neill, 2008). As a result of the protection of unsaturated fatty acids in the fat content of the diet with the addition of protected fat by Ca soap, it may pass from the rumen to the small intestine and muscle tissues at a high rate without undergoing hydrolysis and biohydrogenation processes.

Nutritionists have also focused on the dietary balance between n-3 and n-6 fatty acids formed from PUFAs, such as ALA and linoleic acid. It is preferred that the ratio of n-6/n-3 is lower than 4 because it causes adverse effects on cardiovascular diseases, cancer, inflammation, and immune systems, especially blood coagulation, leading to heart attack (Ruxton et al., 2007). In the study, it was observed that this ratio decreased and approached the desired level with the addition of protected fat ( p<0.05 ), mostly due to the significant linear decrease in the n-6 ratio ( p<0.01 ). However, there was no significantly increase in the total n-3 fatty acid ratio since the protected fat addition did not affect the main n-3 fatty acids in Tuj lambs.

The values of the control group exceeded desirable ranges for the TI and the AI. However, this study determined that the addition of protected fat reduced ( p<0.01 ) AI and TI levels, bringing them close to the upper limit ( AI<1.0 ; TI<1.3 ) specified by Quiñones et al. (2019). The obtained low index values in this study align with the values required for increased protection against coronary diseases. A linear increase in $\Delta^{9}$ -desaturase C18 activity was observed with the addition of protected fat. It is known that the $\Delta^{9}$ -desaturase C18 is involved in the conversion of C18:1 to CLA in the tissues of ruminants (Alves da Costa et al., 2020). A h / H ratio of less than 2.0 is considered optimal for promoting positive modulation of cholesterol transport mechanisms by lipoproteins and preventing cardiovascular diseases (Alba et al., 2021).

## 5 Conclusions

The intake of different levels of protected fat had a significant effect on some important fatty acids, especially on the total amounts of fatty acids, in Tuj lamb meat. The addition of protected fat resulted in a 104 % increase in CLAs (c9, t11) and a 39 % decrease in myristic acid. Similarly, an increase in MUFAs of 20 % and a decrease in SFAs of 11 % were observed with the addition of protected fat.

Meat obtained from lambs fed rations supplemented with protected fat showed improved nutritional properties due to significant positive changes observed in fatty acid composition, including CLA, SFA, n-6/n-3 ratio, and AI and TI indexes. Moreover, no adverse effects were obtained in the physical, chemical, or sensory traits. The results also showed that the enrichment of lamb diets with protected fat from calcium soap enhanced the fattening performance and carcass traits and also provides economic advancement.

The results of the study could provide a contribution to the production of healthy and cost-effective lamb meat. Further research is necessary to determine the effect of adding protected fat to pasture feed on meat quality in young lambs.

## Data Availability

The original data used in this study are available from the corresponding author upon request.
